# Goal sharing with others modulates the sense of agency and motor accuracy in social contexts

**DOI:** 10.1371/journal.pone.0246561

**Published:** 2021-02-04

**Authors:** Kazuki Hayashida, Yuki Nishi, Michihiro Osumi, Satoshi Nobusako, Shu Morioka

**Affiliations:** 1 Department of Neurorehabilitation, Graduate School of Health Sciences, Kio University, Umaminaka, Koryo, Kitakatsuragi-gun, Nara, Japan; 2 Department of Rehabilitation, Fujiikai Rehabilitation Hospital, Yayoi-cho, Higashiosaka-City, Osaka, Japan; 3 Neurorehabilitation Research Center, Kio University, Umaminaka, Koryo, Kitakatsuragi-Gun, Nara, Japan; University of Bath, UNITED KINGDOM

## Abstract

Sense of agency (SoA), the feeling of control over one’s own actions and their effects, is fundamental to goal-directed actions at the individual level and may constitute a cornerstone of everyday life, including cooperative behavior (i.e., goal sharing). Previous studies have demonstrated that goal sharing can activate the motor prediction of both agent’s action and partner’s action in joint-action tasks. Moreover, given that from an SoA perspective, predictive processes are an essential basis, there is a possibility that goal sharing may modulate SoA. However, the possibility for goal sharing to modulate SoA remains unclear. This study aimed to investigate whether goal sharing modulates the intentional binding (IB) effect (a method that can quantitatively measure SoA) of self-generated and observed partner’s actions and improves motor accuracy. Participants were required to stop a circular horizontal moving object by pressing a key when the object reaches the center of a target in a social situation. This task measured IB by having participants estimate the time interval between action and effect in several 100 milliseconds, with shorter time interval estimations indicating enhancement of SoA. Participants were randomly divided into 13 Cooperative groups (goal sharing) and 13 Independent groups (non-goal sharing). Cooperative groups were instructed to perform the task together, while Independent groups did so individually. Participants estimated the time interval between them by pressing the key and hearing the corresponding sound (Self-generated action) and the other person pressing the key and hearing the sound (Observed action). Our results indicated that goal sharing improved motor accuracy and enhanced both the IB of Self-generated and Observed actions compared to non-goal sharing. We suggest that SoA can be modulated by goal sharing in specific social contexts.

## Introduction

Sense of agency (SoA), the feeling of control over one’s own actions and their effects, is fundamental to goal-directed actions at the individual and social levels, being a cornerstone of everyday life in society. SoA is hypothesized to involve predictive processes. At the individual level, a model of action awareness suggests that SoA arises through internal predictions made within a forward action model; this was first proposed as an internal model of motor control and subsequently also considered as a model of SoA, according to which SoA is generated when the predicted effect of the action matches the actual effect [1–6]. Kilner et al. [7] suggested that observed goal-directed actions are processed using the forward model as self-generated goal-directed actions. The forward model may also be useful in explaining the social aspects of SoA.

In social situations, cooperation with others, in which individuals share a common goal according to predefined rules (e.g., a tennis doubles match), can affect the representation of other agents [8, 9]. Joint-action, defined by Sebanz, Bekkering, and Knoblich as a social interaction whereby individuals coordinate their actions in space and time to bring about change in the environment [10], is concerned with studies regarding the processes underlying human cooperative behavior. Pesquita et al. [11] proposed a hierarchical predictive framework for the study of joint action—predictive joint action model (PJAM)—composed of three levels. First, the goal representation level is responsible for maintaining and updating shared goals that guide the interaction. Goal sharing means that both actors have joint goals, are responsive to the intentions and actions of one another, and are committed to the joint activity and mutual support [12]. Second, the action-planning level outputs motor commands that consider both the desired contributions of individuals and their partners to the interaction. Third, the sensory routing level receives the inflow of sensory input and compares it to internal model predictions pertaining to each partner’s action outcomes within the interaction. The PJAM assumes that each agent in a joint action maintains internal models of both themselves and their partners, and each level generates predictions of the information that it expects to find on the level below.

Several previous studies indicate that joint action can modulate the internal forward model. Sacheli et al. [13] investigated the cognitive processes that underpin motor interactions on joint action. They tested participants who took turns playing music with a virtual partner and their results demonstrated that joint action was based on active prediction of the partner’s action effects rather than on passive action imitation. These findings suggest that such predictions are based on Dyadic Motor Plans that represent both the agent’s and the partner’s contributions to the interaction goal. In addition, Sacheli et al. [14] reported that left ventral premotor cortex activity demonstrates that their participants process the partners’ behavior to prospectively infer their contribution to the shared goal achievement, generating motor predictions for cooperation beyond low-level imitation. In their electroencephalography study Kourtis et al. [15] also provided evidence that when people engage in joint tasks, they represented each other’s actions in advance to facilitate coordination. Furthermore, Kourtis et al. [16] showed that engaging in joint action formed sensorimotor representations. The activation of this primary sensorimotor areas contralateral to the acting hand suggests that goal sharing is related not only in the partner’s action planning but also in the agent’s action execution.

These essential studies provide evidence that goal sharing can activate the dyadic motor prediction of both agent’s action and partner’s action. Since an internal forward model is also an essential basis from an SoA perspective, there is a possibility that goal sharing may modulate SoA. It may be reasonable to assume that SoA is instrumental for successful joint action since it allows for conscious top-down modulations regarding how a shared goal may be broken down into individual contributions [11]. In a previous study, participants were paired for a joint action task in which one person acted to generate an outcome, while the other acted thereafter [17]. The results showed that both participants demonstrated the same levels of SoA. This pattern of results supports the idea that partners process the link between each other’s actions and sensory outcomes. However, this suggests that this process alone does not lead to the goal representation level and the possibility for goal sharing to modulate SoA remains unclear.

To investigate this possibility, it is necessary to quantitatively measure SoA and performance using experiments wherein participants achieve a certain task in cooperation with others. We had to first perform a motor task toward a goal at the individual level and examine its effect on SoA [18]. We recently reported a method that measures motor accuracy and the intentional binding (IB) effect (see below), which can quantitatively measure SoA [18]. In particular, we developed a motor performance task that required stopping a circular horizontal moving object by pressing a key when the object reaches the center of a target and reporting the relation between perceptual-motor accuracy and the IB effect. Our previous study proposed that using a motor task to achieve strong goals enhanced SoA [18].

IB is often used to measure SoA quantitatively without explicit judgments. In recent years, IB has been reported as a causal inference of action and effect in agency [19, 20]. A method used to measure the IB effect involves estimating the time interval between pressing a key and hearing an auditory stimulus, which directly measures the shift in the perceived times of the action and its effect [19, 21, 22]. The perceived shorter time interval is used as an index of a greater IB effect. Moreover, measuring the IB effect can reveal various factors that modify SoA. Poonian et al. [23] demonstrated the IB effect by observing others’ actions; in particular, participants estimated the time interval between an observed action and a tone as significantly shorter than the time interval between two tones. As no significant difference was found between self-generated action and the other action conditions, these results suggest that the observation of another agent’s intentional goal-directed action elicits IB. In a previous study, it was found that since the prediction process modulates IB [24], IB involves a forward model. Considering that IB is also influenced by joint action [17], applying the IB task may be useful in investigating the modulation of the internal forward model of each partner with goal sharing.

In this study, we developed a social version of our previous experiment [18] that included IB and motor performance tasks and explored whether goal sharing could affect them. In the current experiment, participants were divided into Cooperative groups (goal sharing) and Independent groups (non-goal sharing) and were required to take turns playing the motor performance task with a partner. Goal sharing may activate motor predictions in both the agent and partner, and not just one or the other since joint actions interact with agents and partners. Thus, we measured the IB of self-generated and observed actions. This study aimed to investigate whether goal sharing modulates the IB both self-generated and observed actions and improves motor accuracy. We expected that goal sharing would enhance the IB effects of both self-generated and observed actions, not just one or the other, and would improve motor accuracy more than non-sharing goal. This study may expand the understanding of social-level mechanisms in the internal forward model of SoA.

## Materials and methods

### Participants

The participants were 26 right-handed, healthy, same-gender pairs. We divided the participants by pair into a Cooperative group (13 pairs; 18 females, *mean* age = 20.8, *SD* = 0.8) and an Independent group (13 pairs; 20 females, *mean* age = 20.2, *SD* = 1.1). All participants reported the normal vision, hearing, and verbal and finger function needed for the experiment. The Kio University ethics committee approved the study’s procedures (H28-50), and the experiment was conducted in accordance with the Declaration of Helsinki. All participants provided written informed consent prior to their participation in the experiment.

### Materials

The task and measuring system were created using Laboratory Virtual Instrument Engineering Workbench (National Instruments). A 19-inch display (Mitsubishi RDT191VM, Japan) and two keyboards (DELL RT7D60 Microsoft comfort curve keyboard 3000, Japan) were used to conduct the experimental task.

### Apparatus and procedure

**Fig 1:**
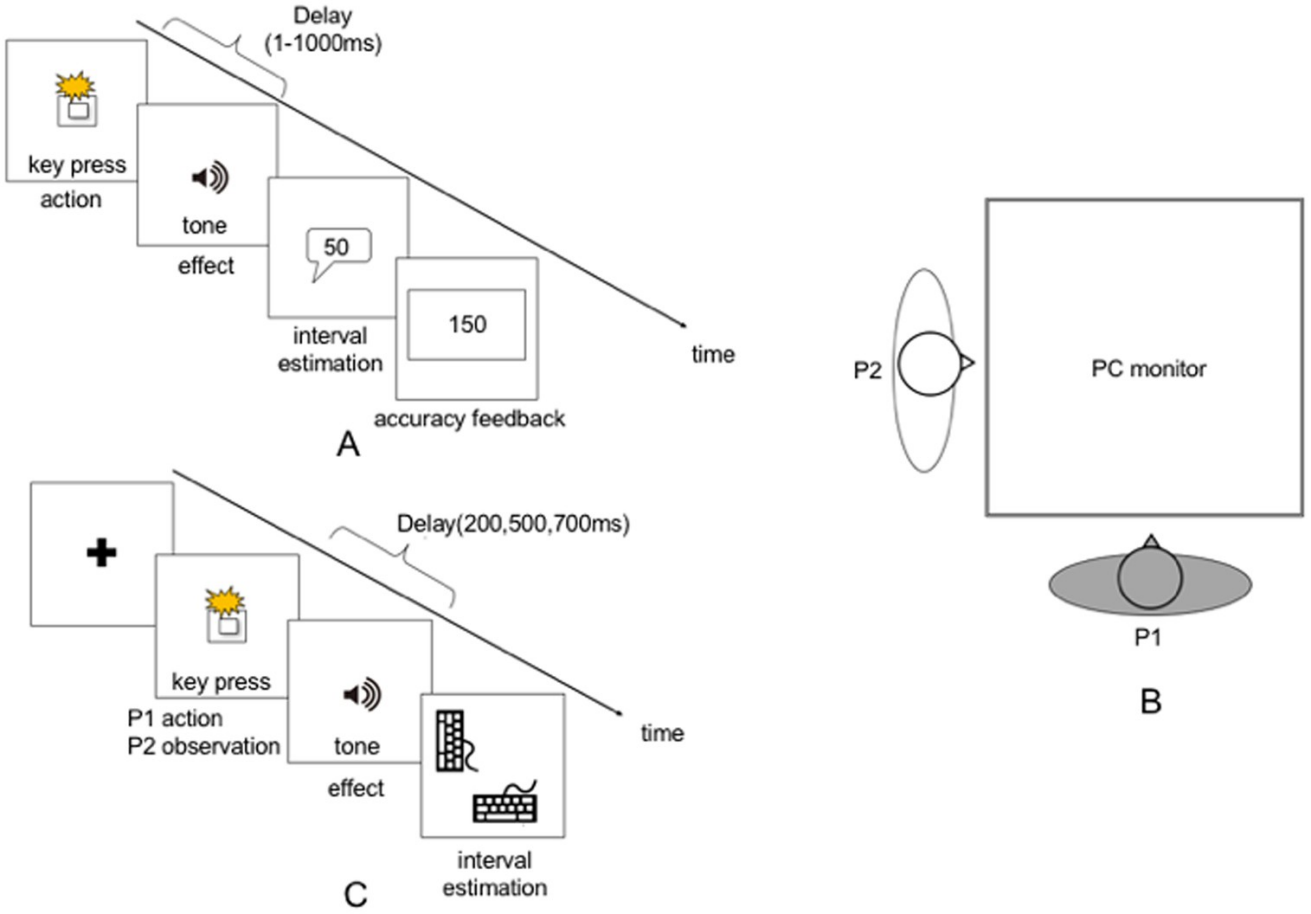
A. Training task. Participants first individually completed a preliminary practice exercise to familiarize themselves with the task setup. To practice estimating the time interval after pressing a key with the right index finger, there was a delay of 1–1000 ms before a tone sounded (50 ms, 900 Hz). Participants then estimated the time interval orally. Different intervals were randomly selected for each trial, and after responding, each participant received feedback on the actual interval. B. Sitting position in the baseline and experimental tasks. We placed the pairs horizontally in front of a monitor. P = participant. C. Baseline task. After the key press, a delayed tone sounded. The actor and the observer, respectively, estimated the delay intervals. Participants answered using a keyboard instead of verbally, and participants alternated key pressing and observation in each of the 18 trials. P = participant https://doi.org/10.6084/m9.figshare.9755408.v1.

Participants first individually completed a preliminary practice exercise to familiarize themselves with the experimental task’s set up. The time interval between a key press with the right index finger and a tone sounding (50 ms, 900 Hz) was 1–1000 ms. In ms units, participants verbally estimated the time interval between the key press and the tone. This preliminary task was administered to participants over 18 trials. The time intervals were random for each trial, and after responding, each participant received feedback on the actual interval (Fig 1A). Because this preliminary task’s purpose was participant training, it was excluded from subsequent analyses.

When building the experimental model, we speculated that there would be a large variation in the perceived time of <1 s, depending on the individual. For example, a value of time awareness of 100 ms would vary between individuals who perceive 100 ms as 200 ms and individuals who perceive 100 ms as 400 ms; we thought that this individual difference would affect IB. Therefore, a baseline task was conducted to correct individual bias on temporal perception because this experimental design includes a between-subjects factor. Participants sat in pairs in front of a monitor (Fig 1B). After a black cross was presented onscreen for 1 s, one member of the pair pressed a key at an arbitrary timing, and the other observed the key press. Each participant (key presser and observer) estimated the time interval from the key press (action and action observation) to the tone. Experimental time intervals were, randomly, 200, 500, or 700 ms, but participants knew only that the interval was random, from 1 to 1000 ms. At this point, participants received no feedback on the actual time interval, and their interval estimates were reported by key pressing instead of orally. The partners did not see each other’s estimations and were unable to know the other’s estimation of the time interval. One by one, pairs alternated key pressing and action observation for 18 trials (Fig 1C). We referred to the literature [18, 25] to determine the appropriate number of trials required for our study.

In the experimental task, participants performed an IB task that included a perceptual-motor task and elements of goal sharing. We referred to our previous research [18] while developing the task for the experiment. Participants were first presented with a black fixation cross for 1 s; next, a red, circular, flat object repeatedly moved horizontally across the screen (1.5 times per second) at a constant speed (3,294 px/s), and participants pressed a key when the object reached the screen’s center. The circular object had a radius of 20 px, and the circle in the center of the screen had a radius of 30 px. The size of the screen was 630 × 630 px. The distance of the error (px) between the object’s center and the target’s center was calculated, with lower error values indicating higher temporal precision of motor performance. The object stopped moving as soon as the participant pressed the key, and after a delay, a tone was presented. The participant estimated the interval between pressing the key and hearing the tone. As in the baseline task, the observer also estimated the time interval. The difference between the estimated time interval in the baseline task (key press at an arbitrary time) and that in the experimental task was calculated.

**Fig 2:**
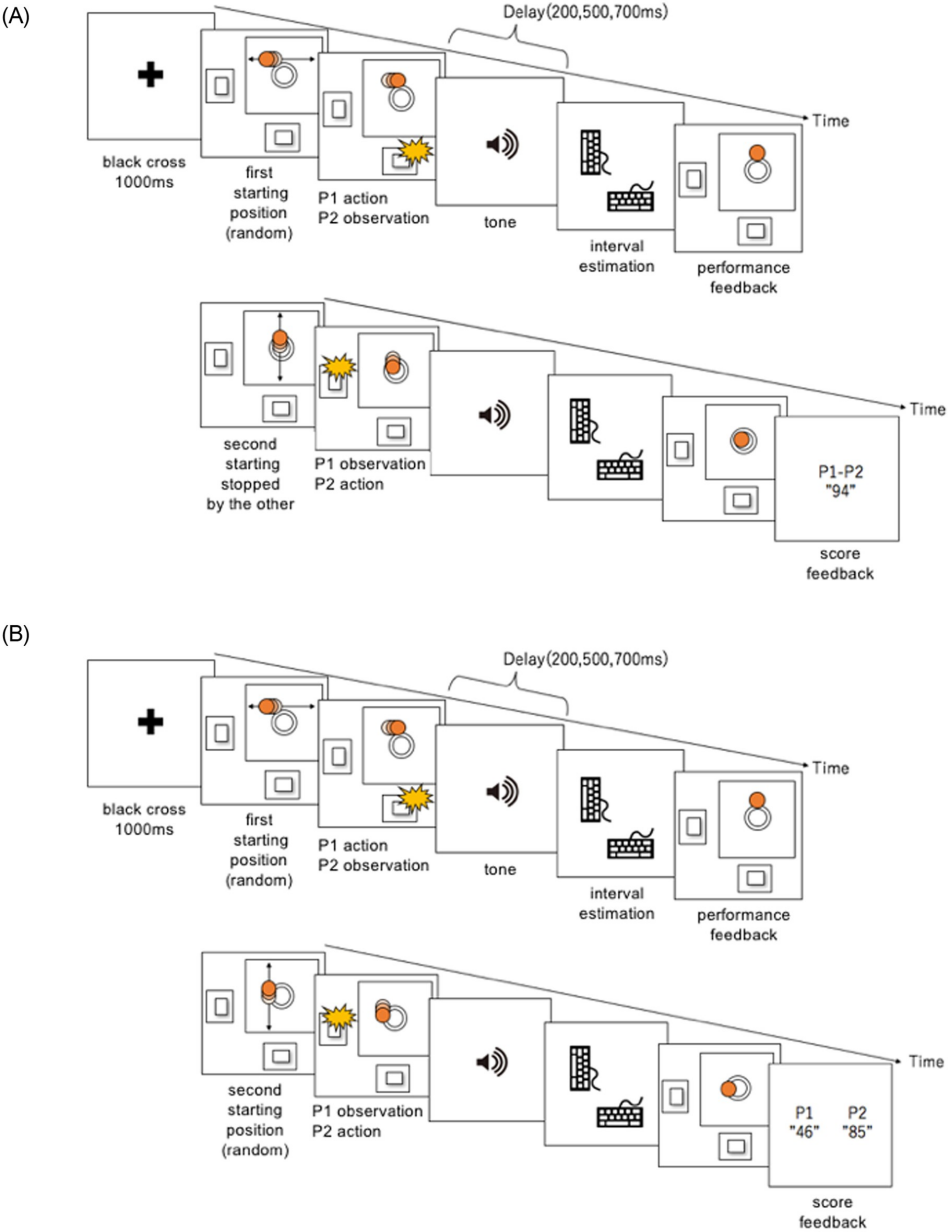
A. Experimental task—Cooperative group. Participants shared goals with others and cooperated to score points. The object’s first starting position was random, but its second starting position was from where the first participant stopped it. B. Independent group. Pairs were not asked to share, and each scored points separately. The object’s first and second starting positions were random. P = participant https://doi.org/10.6084/m9.figshare.9755408.v1.

In the Cooperative group, the object’s onscreen starting position was random for the first participant, who then stopped the object’s motion by pressing a key. Next, the object began to move vertically and repeatedly from the position where the first participant had stopped it, and the second participant attempted to stop the object at the screen’s center by pressing a key. In the Cooperative group, the position where the second participant stopped the object was presented onscreen as the cooperative result. When an error between the object’s stop position and the onscreen center was zero, 100 points were awarded. Motor accuracy results were presented for each trial. For cooperative pairs, their total points were presented, and participants were instructed to cooperate (Fig 2A). For the independent pairs, the object’s onscreen starting position was random for the first and second participants, and the first participant’s result did not affect the second participant’s task (Fig 2B). Independent pairs were instructed to aim for 100 points per participant, and each participant’s score was displayed individually. The perception of the time interval was taken immediately after the tone sounding and before the performance feedback. The same procedure was used for the Cooperative and Independent groups. For each trial, participants alternated between the first (action) and the second (action observation). This task comprised 10 blocks (18 trials/block), and the response method for the time interval and the interval estimation was the same as in the baseline task.

### Statistical analyses

Perception of the time interval varied per participant. For the experimental task, the mean interval value of the baseline task set of Self-generated and Observed action trials was subtracted from each set’s IB value (in Self-generated action [or Observed action] in the experimental task, estimated interval − actual interval) − (in Self-generated action [or Observed action] in the baseline task, estimated interval − actual interval). This calculation followed another study [18]. Analysis using the Kolmogorov–Smirnov test showed that all the mean data of the IB and motor accuracy were normally distributed. Thus, we analyzed the IB effect using a mixed within-between ANOVA with “group” (i.e., Cooperative and Independent) as between factor and “action” (i.e., Self-generated and Observed) as within-subject factor.

In the previous study [18], we confirmed the characteristics of the experiment of the time-series floor effect. To investigate the effect and the influence on motor accuracy in goal sharing, we analyzed the changes over time. To avoid the type II error, the 10 blocks were divided into the early phase (1–3 blocks), middle phase (4–6 blocks), and last phase (7–10 blocks). Motor accuracy was analyzed using two-way mixed ANOVA that accounted for “group” (i.e., Cooperative and Independent) as between factor and “phase” (Early, Middle, and Last) as within-subject factor. The Bonferroni method was used for post-hoc comparisons.

The results of the first participants cumulated points in the Cooperative group but not in the Independent group. Thus, in additional analysis, we analyzed the effect of order bias by pressing the key between groups. Motor accuracy was analyzed using two-way mixed ANOVA that accounted for “group” (Cooperative and Independent) as between factor and “order” (First and Second) as within-subject factor.

Data regarding the intentional binding effect and error and order effect of motor accuracy are shown in S1 Table. *P* values <0.05 were considered statistically significant. SPSS Statistics for Windows ver. 24 (IBM, Japan) was used as the analysis software.

## Results

**Fig 3:**
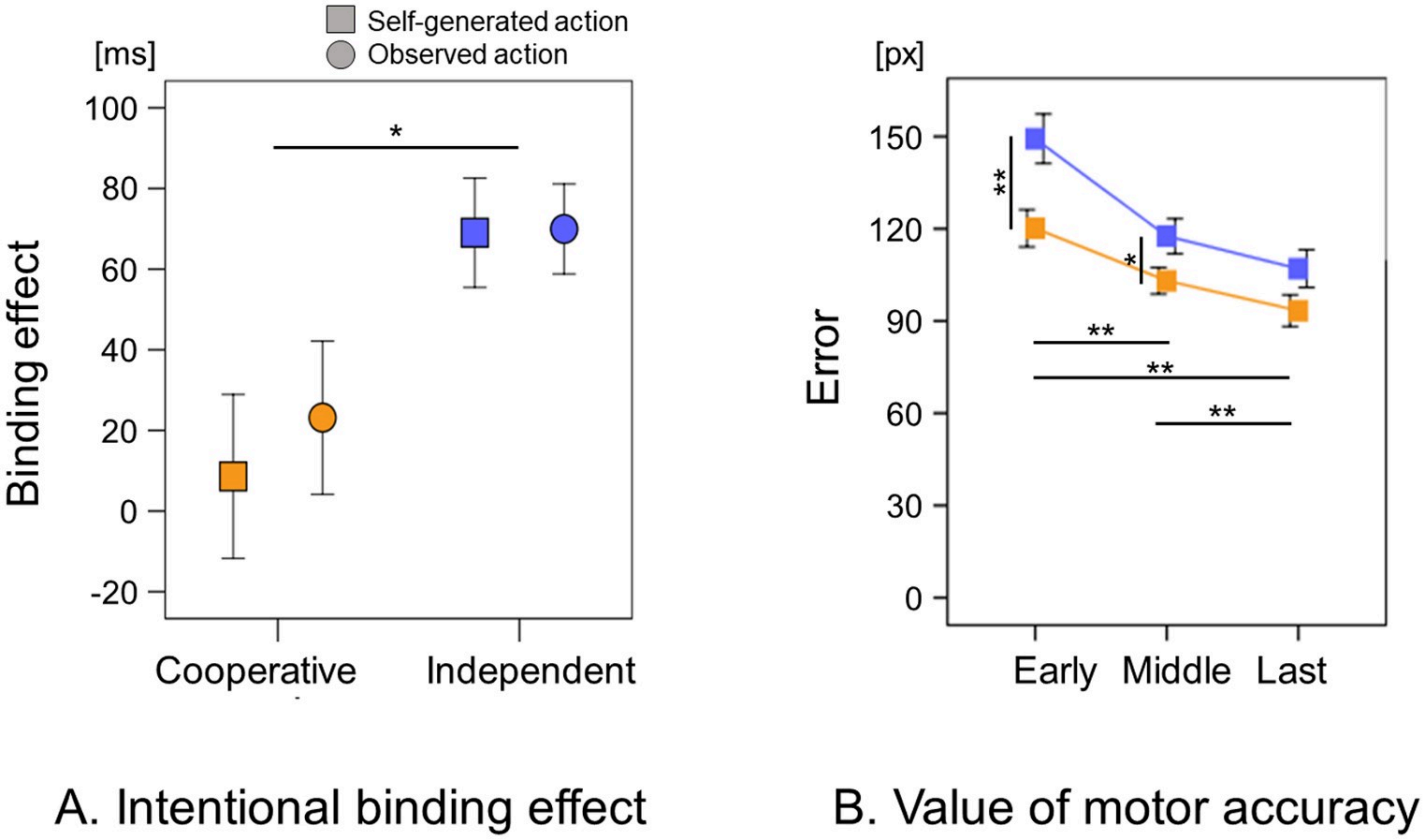
A. Intentional binding effect by Self-generated and Observed action. Yellow plot: Cooperative group; blue plot: Independent group; Square plot: Self-generated action; Round plot: Observed action. B. Value of motor accuracy. Yellow plot: Cooperative group; blue plot: Independent group. Data represent means ± standard error. * indicates a *p*-value < 0.05. ** indicates a *p*-value < 0.01. https://figshare.com/articles/figure/Figure_3_/12923612.

A lower IB value indicates a higher SoA. The interaction was not significant (*F* (1,50) = 0.838, *p* = 0.364, *η*_*p*_^*2*^ = 0.016). The main effect of group was significant (*F* (1,50) = 5.926, *p* = 0.019, *η*_*p*_^*2*^ = 0.106), with lower IB value for the Cooperative group than for the Independent group. The main effect of action was not significant (*F* (1,50) = 1.081, *p* = 0.303, *η*_*p*_^*2*^ = 0.021) (Fig 3A). S2 Fig shows the estimated time interval in each actual time interval.

The distance of the error (px) between the object’s center and the target’s center was calculated, with lower error values indicating higher motor accuracy. The result of the error showed a main effect in the group factor (*F*(1, 50) = 6.48; *p* = 0.014), phase factor (*F*(2, 100) = 60.76; *p* < 0.001) and interaction (*F*(2, 100) = 3.72; *p* = 0.028). The error in the Early phase was significantly higher than the error in the Middle phase (*mean difference* = 23.24, *95%CI* = 14.64–31.84, *p* < 0.001) and in the Last phase (*mean difference* = 35.44, *95%CI* = 26.66–44.23, *p* < 0.001). The error in the Middle phase was significantly higher than that in the Last phase (*mean difference* = 12.20, *95%CI* = 5.472–18.93, *p* < 0.001). The error in the Cooperative group was significantly lower than that in the Independent group in the Early phase (*mean difference* = −28.86, *95%CI* = −48.60 − −9.12, *p* = 0.005) and in the Middle phase (*mean difference* = −16.50, *95%CI* = −31.90 − −1.09, *p* = 0.036). No significant differences were observed in the error of both groups in the Last phase (*mean difference* = −11.54, *95%CI* = −26.28 − −3.19, *p* = 0.12) (Fig 3B).

In the additional analysis, the interaction was not significant (*F* (1,50) = 0.335, *p* = 0.556, *η*_*p*_^*2*^ = 0.007). The main effect of the group was significant (*F* (1,50) = 11.018, *p* = 0.002, *η*_*p*_^*2*^ = 0.181), with lower error in the Cooperative group than the Independent group. However, the main effect of order was not significant (*F* (1,50) = 3.497, *p* = 0.067, *η*_*p*_^*2*^ = 0.065) (S1 Fig).

## Discussion

This study examined the influence of goal sharing on IB and motor accuracy. We developed a novel IB task that aimed to test whether goal sharing can modulate both motor accuracy and SoA, which was examined quantitatively. In this task, participants predicted when the repetitively moving object will reach the center of the screen and then pressed the key at their desired time. We posit that this task represented the decision-making process regarding the timing of action initiation based on the visual information of the internal model. Participants were divided into goal sharing and non-goal sharing groups and were required to estimate the time interval of the Self-generated or Observed actions. We also confirmed that goal sharing improved motor accuracy and strongly enhanced the IB effects on both Self-generated and Observed actions compared to non-goal sharing. Our findings suggest that the forward model is also useful in explaining the impact of goal sharing on the SoA.

In the Cooperative group, participants were required to attempt to achieve two goals: (1) an explicit goal of stopping the object at the target and (2) a high-level goal of improving each other’s motor performance. The higher motor accuracy in the Early and Middle phase of the Cooperative group compared to the Independent group may suggest that goal representation level affects the internal model. No significant difference in motor accuracy in the Last phase may indicate the floor effect. We have to consider one concern that could affect the results of motor accuracy. Specifically, in the Cooperative group, the stopping position of the first participant was accumulated and the second participant needed to improve the first participant’s performance. In contrast, in the Independent group, the performance of the first participant did not affect the second participant. Therefore, we should be careful about strong interpretations. Additional analysis confirmed that there was no effect of order. Moreover, we believe that there was no statistical advantage between groups because there was no significant difference in the Last phase. If accumulation could affect the results, there should be differences between groups in the Last phase as well. From these, we believe that the cumulative effect of order did not affect the results, but the effect of goal sharing.

Importantly, enhancement of the IB effect of Observed action in the Cooperative group compared to the Independent group may support the framework of PJAM. This result supports evidence from previous studies indicating that active observations for cooperation, rather than passive imitation, activated motor prediction [13]. In addition, a previous study suggested that the common aspect in self-generated and observed actions was a prediction of the internal model [26]. In the present task, pairs alternated between Self-generated action and Observed action in every trial. The IB of Observed action could have represented a motor code equivalent to another’s action [27], which may have been activated by goal sharing.

Enhancement of the IB effect of not only Observed action but also Self-generated action in the Cooperative group compared to the Independent group may support our hypothesis that goal sharing modulates SoA. However, the PJAM does no suffice in explaining the effect of goal sharing on IB that we found in the Self-generated action condition. The responsibilities created by sharing goals may have affected attention and influenced the IB effect [28]. The main difference between the groups in this study was not whether they shared the action [17] but whether they “shared the goal.” Wenke et al. [29] suggested that the partial responsibility of a co-actor affects co-representation in task-sharing situations, such as table tennis. In addition, a sense of responsibility enhances SoA [30, 31]; therefore, a sense of responsibility created by goal sharing may have influenced the IB effect of Self-generated action in the Cooperative group. This result suggested that the internal model is modulated merely by sharing the representation of a goal rather than sharing the action. In contrast, in the Independent group, the display of individual scores might have instigated competition among participants. Although competition and cooperation can affect performance, few researchers have investigated the difference between the effects of competition and cooperation on IB and motor accuracy [32]. Considering the relevance of social contexts other than joint action, verification of how effective goal sharing is for the internal model of SoA is necessary. These concerns may better explain the impact of goal sharing on SoA.

One notable point should be considered regarding the IB effect, which was a positive value. The IB effects of the results were subtracted from the baseline task, and a positive value indicated that the IB effect was smaller in the experimental task. We have two explanations for this. One is the timing of the key pressing; studies have suggested that limiting the timing of action selection reduced the IB effect [33]. In the baseline task, participants were required to press a key at a self-paced rate without the limitation of timing. In the experimental task, the key-press timing was extremely limited by the object reaching the target. Therefore, the IB effect may be smaller because the experimental task was more time limiting than that in the baseline task. However, the timing of the action is not forced externally (e.g., a hitter swinging at a baseball) but is partly the result of free timing. Thus, further research should control the degree of freedom of time to verify how it affects IB. Second, an alternative explanation is that participants are required to engage in motor accuracy in the experimental task compared with the baseline task, and the ability to pay attention may have affected their temporal perception [28]. These explanations probably influenced the value of the IB effect in the experimental task.

An ideal situation such as in the classic paradigm is to incorporate spatial elements into the experimental task when observing motor performance. However, the IB-related tasks developed in other studies were almost exclusively concerned with index finger movement, and we posit that the experimental paradigm used in Imamizu et al. [34] is inappropriate if the goal is to observe an IB effect. Therefore, following other IB-related studies, we set up a task without spatial elements. Further research should include tasks for performing interactively with others, including temporal and spatial parameters, to study them in more detail.

Notably, one of the limitations of this study is that it is possible that the elements of competition were introduced in the Independent groups. Even if the Independent group was affected by competition, the significant difference in the IB effect is a crucial finding. Hence, further research should examine the effects of competition and cooperation. Furthermore, the lack of control over baseline motor accuracy is a concern. Our previous study has confirmed that a few participants had low motor performance [18]. If controlled by the baseline task of motor accuracy, the results may have been more accurate. In addition, since the performance of the first participants influences the second participants, our study may have limited applicability. Another limitation was that the bias of two-part relationships occurred because this experiment involved pairs. It is necessary to explore how the influence of others affects IB and motor accuracy by measuring compatibility and interpersonal skills.

## Conclusions

We arranged a novel social version of an IB task and examined the influence of goal sharing on IB and motor accuracy. It was found that sharing a goal with another individual resulted in improved motor accuracy and enhanced IB effects of self-generated and observed action compared to non-goal sharing. Hence, we suggest that SoA can be modulated by goal sharing in specific social contexts.

## Supporting information

S1 Table The data of the intentional binding effect and error. https://figshare.com/articles/dataset/Table/13615358.
(XLSX)

S1 Fig Order effect of motor accuracy. https://figshare.com/s/37ac2c0d46738d5660b6.
(TIF)

S2 Fig The estimated time interval in each actual time interval. https://figshare.com/articles/figure/S3_Figure/13615400.
(TIF)
