# Supplementary figures and images for: Goal sharing with others modulates the sense of agency and motor accuracy in social contexts
Source: PLoS One. 2021 Feb 4;16(2):e0246561. doi: 10.1371/journal.pone.0246561 (PMC7861436; doi:10.1371/journal.pone.0246561)

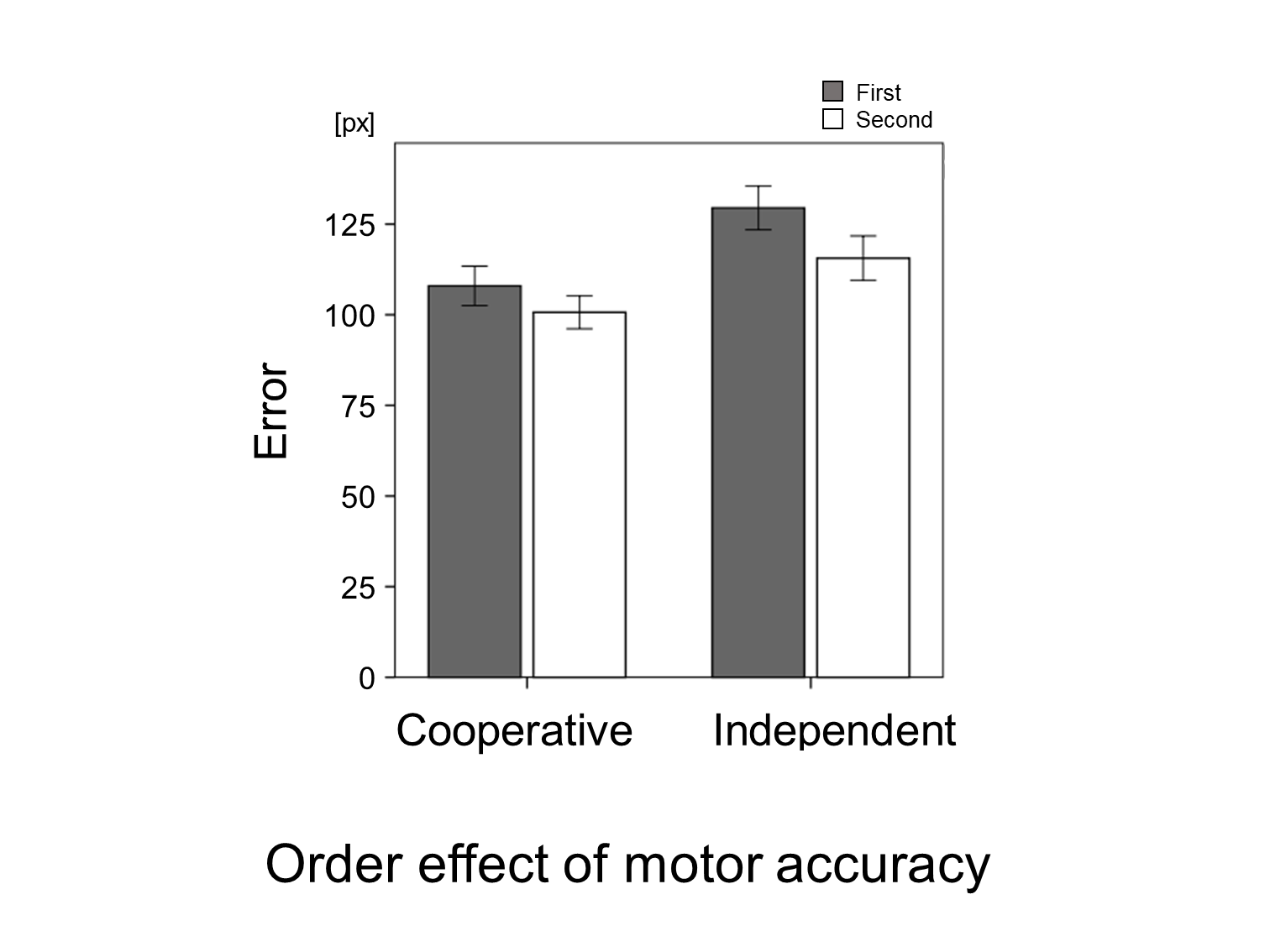

Supplement: S1 Fig — https://figshare.com/s/37ac2c0d46738d5660b6. (TIF) [file pone.0246561.s002.tif]

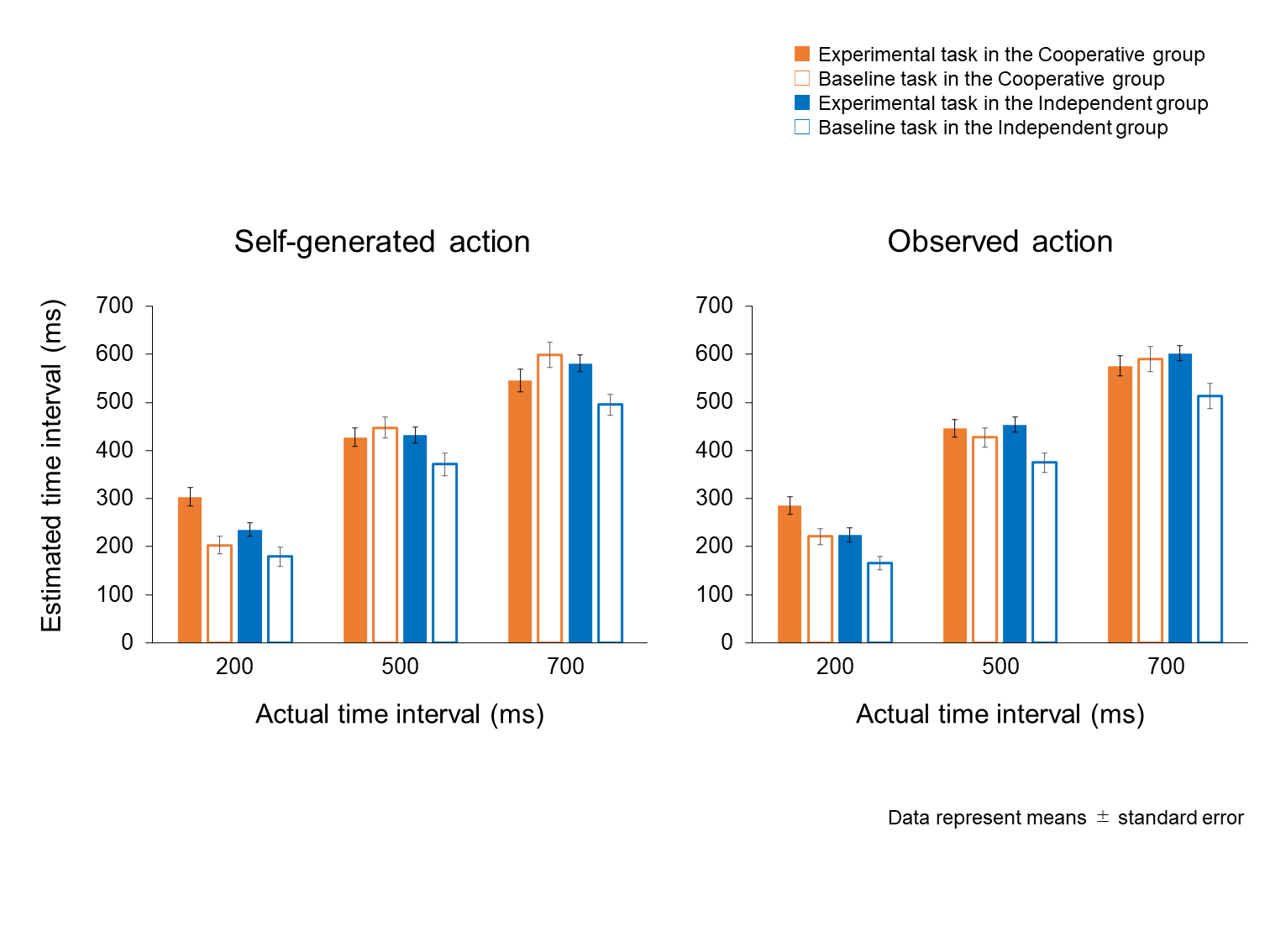

Supplement: S2 Fig — https://figshare.com/articles/figure/S3_Figure/13615400. (TIF) [file pone.0246561.s003.tif]
